# A Novel Method for Hand Movement Recognition Based on Wavelet Packet Transform and Principal Component Analysis with Surface Electromyogram

**DOI:** 10.1155/2022/8125186

**Published:** 2022-11-08

**Authors:** Yingda Huo, Fubao Li, Qin Li, Enqiu He, Jichi Chen

**Affiliations:** ^1^School of Mechanical Engineering, Shenyang University of Technology, Shenyang 110870, Liaoning, China; ^2^School of Chemical Equipment, Shenyang University of Technology, Liaoyang 111003, Liaoning, China

## Abstract

As an input method of signal language, the hand movement classification technology has developed into one of the ways of natural human-computer interaction. The surface electromyogram (sEMG) signal contains abundant human movement information and has significant advantages as the input signal of human-computer interaction. However, how to effectively extract components from sEMG signals to improve the accuracy of hand motion classification is a difficult problem. Therefore, this work proposes a novel method based on wavelet packet transform (WPT) and principal component analysis (PCA) to classify six kinds of hand motions. The method applies WPT to decompose the sEMG signal into multiple sub-band signals. To efficiently extract the intrinsic components of the sEMG signal, the classification performance of different wavelet packet basis functions is evaluated. The PCA algorithm is used to reduce the dimension of the feature space composed of the features reflecting hand motions extracted from each sub-band signal. Besides, to ensure higher classification performance while reducing the dimension of the feature space by the PCA algorithm, the classification performance of different dimensions of the feature space is compared. In addition, the effects of the variability of the sEMG signal and the size of the window on the proposed method are further analyzed. The proposed method was tested on the sEMG for Basic Hand Movements Data Set and achieved an average accuracy of 96.03%. Compared with the existing research, the proposed method has better classification performance, which indicates that the research results can be applied to the fields of exoskeleton robot, rehabilitation training, and intelligent prosthesis.

## 1. Introduction

Hand movements are the most meaningful and elementary form of human daily communication and represent the intentions expressed by people [1, 2]. As a signal language input method, hand movement classification has important theoretical research significance and practical application value in the field of human-computer interaction [3]. As a result, hand movement classification technology that allows humans to communicate with computers more efficiently, conveniently, and naturally has developed into an important part of the field of artificial intelligence [4, 5]. Many studies have classified hand movements in terms of computer vision and wearable sensors [6–8]. Compared with computer vision, sEMG signals collected by wearable sensors are an ideal source for hand motion classification [9, 10]. Although there are many studies using sEMG signals for hand motion classification, how to extract the effective components of sEMG signals to achieve accurate hand motion classification is still a challenging problem.

According to the method of collecting data, hand movements classification can be divided into two categories: hand movement classification based on computer vision and hand movement classification based on wearable sensors [2]. Based on computer vision hand movement classification, hand movement images are captured by the camera and then the feature is obtained through image processing technology to classify the hand movements [6]. Sharma et al. [7] used a convolutional neural network to recognize images of Indian sign language gestures collected with an RGB camera. Kumar et al. [11] proposed two viewpoint-set-up gesture classification methods. Their experimental results show that compared with a single-camera system, this method has high classification accuracy even when simple classifiers such as nearest neighbors and decision trees are used. However, the performance of hand movement classification based on computer vision is often affected by factors such as light intensity, shooting distance, shooting angle, and occlusion of sight [12]. Hand movement classification based on wearable sensors overcomes the above-mentioned problems by detecting signals generated by hand movements. Jiang et al. [13] designed a real-time gesture classification wristband based on the sensor fusion of sEMG and inertial measurement unit. Their initial experimental results show that the classification accuracy of air and surface gestures is 92.6% and 88.8%, respectively. Wearable sensors for hand movement classification include accelerators, inertial measurement units, gyroscopes, and sEMG signal sensors [14–17]. Compared with the signals recorded by other wearable sensors, the sEMG signal reflects the human body's electrophysiological response to various activities and has inherent advantages in predicting actions and distinguishing between passive and active activities [18]. The sEMG signal has become the primary research approach in the fields of hand movement classification, activity recognition, and gait analysis [19–21].

The sEMG signal is the superposition of action potentials of many motor units in time and space, which reflects the body's movement intention [22, 23]. The sEMG signal is an unstable bioelectric signal with different frequency components at different moments [24]. The representative time-frequency analysis methods of the sEMG signal include fast Fourier transform (FFT) [25], short-term Fourier transform (STFT) [26, 27], Wigner–Ville distribution (WVD) [28, 29] and Hilbert–Huang transform (HHT) [30], and wavelet transform (WT) [18, 31, 32]. The prerequisite for FFT and STFT to effectively analyze the signal is that the signal is stable [33]. It is obvious that they cannot effectively reflect the time-frequency characteristics of nonlinear and nonstationary sEMG signals. Compared with STFT, WVD can interpret the signals better [34]. However, WVD may have cross-terms that carry important information about the relationship between signal components to mask the original signal, making it difficult to interpret the time-frequency information of the signal [35, 36]. The time-frequency analysis of the signal by the HHT method consists of two steps, namely, empirical mode decomposition and the Hilbert transform. The HHT method is suitable for nonlinear and nonstationary signal analysis, but the computational cost is higher than that of WT and other methods [24]. The WT analyzes the local characteristics of the signal at different time periods and in different ranges by calculating the convolution of the signal and the wavelet basis function [37]. The WPT is an important extension of the WT, which can effectively analyze the frequency components of the signal [38]. Existing research has shown that WPT with many resolution levels can effectively extract the components in the signal to obtain better classification results [31, 39].

Feature extraction plays a vital role in the classification of hand movements based on sEMG signals [40]. Feature extraction is to convert the sEMG signal into a compact and information-rich feature space. The sEMG signal feature extraction methods are generally divided into time-domain (TD) features and frequency-domain (FD) features [41]. The TD feature is the TD statistics obtained by directly performing statistical analysis on the signal amplitude [42]. Common TD features include root mean square (RMS), variance (VAR), mean absolute value (MAV), waveform length (WL), and zero crossing (ZC) [43–45]. Arief et al. [46] evaluated five TD features to find the best way to minimize the complexity of implementation and reduce the cost of information processing. The FD features are extracted from the frequency spectrum of the sEMG signal [47]. According to the research of hand movement classification based on sEMG signals, the FD features extracted from sEMG signals mainly include median frequency (MDF), mean frequency (MNF), and mean power (MNP) [41]. Phinyomark et al. [48] proposed two modified FD features for robust feature extraction.

**Figure 1:**
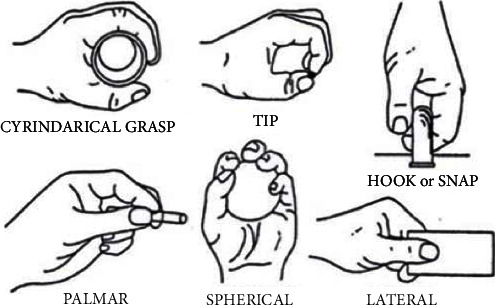
The six corresponding hand movements.

To improve the classification performance of hand movements based on sEMG signals, we propose a novel method for hand movement classification. In this method, the sEMG signal is decomposed into multiple sub-band signals by WPT; the TD and FD features are extracted from the frequency band signals; the PCA is used to eliminate redundant features; and machine learning is used as a predictive model to achieve the purpose of accurately classifying hand movements. The method proposed in this work is of great significance to the development of human-computer interaction, clinical medicine, and prosthetic control. The flow chart of this work is shown in Figure 1.

Based on the above analysis, the motivation of this research is to achieve a high-accuracy hand movement recognition method that can accurately extract effective information from sEMG signals. In this work, we adopt WPT to decompose the sEMG signal into multiple frequency band components to represent the most important information in the sEMG signal. To select a suitable wavelet basis function, we evaluated the classification accuracy of different wavelet packet functions for hand movements. MAV, RMS, MNF, and MDF were extracted from sub-band signals obtained by the WPT decomposition of sEMG signals. The extracted features are projected into a low-dimensional space by PCA to remove unimportant features. Machine learning classifiers are used to recognize hand movements and compare the corresponding recognition performance.

The rest of the work is structured as follows: Section 2 describes the database and methods used in this work in detail.  Section 3 shows the results.  Section 4 discusses the proposed method.  Section 5 presents the conclusion.

## 2. Related Works

The sEMG signal data set used in this work is obtained from the publicly available sites. The sEMG signal contains abundant information on body movements and has great potential for hand motion classification [49]. However, the analysis of sEMG signals is a challenging problem due to the fact that the acquisition of sEMG signals is affected by electrode displacement, muscle structure differences, and muscle contraction strength [50]. Based on this challenging problem, many scholars have conducted in-depth research on the classification of hand movements based on sEMG signals [14, 32].

Nishad et al. [8] apply tunable-Q wavelet transform-based filter-bank (TQWT-FB) for decomposition of cross covariance of sEMG signals. Kraskov entropy features are extracted from each sub-band signal. RELIEFF is used for feature ranking. Features with significant differences are selected and input into the k-NN classifier for hand motion classification. Sapsanis et al. [10] utilized empirical mode decomposition to decompose sEMG signals into intrinsic mode functions (IMFs). Eight features (IEMG, ZC, VAR, SSC, WL, WAMP, kurtosis, and skewness) are extracted from IMFs and raw sEMG signals. A simple linear classifier was used to achieve the classification of the six hand movements. Ruangpaisarn et al. [51] proposed a method based on singular value decomposition and SMO to classify six basic hand movements. The authors propose the V2M-SVD method for feature extraction of sEMG signals, and the SMO classifier is used for the classification of six hand movements. Yavuz et al. [52] proposed a method based on cepstral analysis to classify basic hand movements from sEMG signals. By calculating the mel-frequency cepstral coefficients (MFCCs), the cepstral analysis technique is used to extract the TD features of the sEMG signals. The extracted feature vectors are composed of MFCCs, and then, a generalized regression neural network (GRNN) is used to classify basic hand movements. Fatimah et al. [53] proposed an automatic recognition algorithm for hand movements based on the Fourier decomposition method (FDM). The method adopts FDM to decompose the sEMG signal into Fourier intrinsic band functions (FIBFs). The kurtosis, entropy, and L1 norm of each FIBF are extracted as features and fed into a machine learning classifier to classify hand motion.

According to the research mentioned above, it is a challenging problem to effectively extract intrinsic components from sEMG signals to achieve accurate hand motion classification. Therefore, this work proposes a novel approach to improve the classification performance of hand motions. The contributions of this work are presented as follows:

- The hand motion classification performance of WPT based on different wavelet basis functions is evaluated
- The classification performance of the proposed method based on different feature space dimensions is compared
- The robustness of the proposed method is analyzed
- The classification performance of the proposed method with different window sizes is tested

## 3. Materials and Methods

### 3.1. Data Set

**Figure 2:**
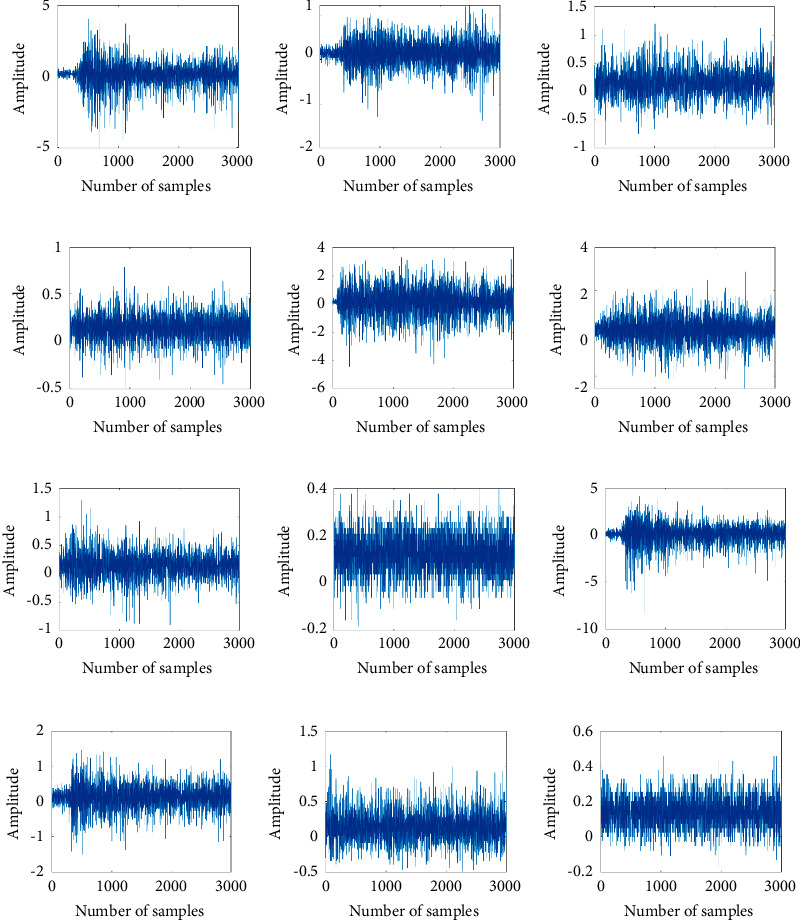
The sEMG signal recorded from female_1: (a) CY hand movement from electrode 1; (b) CY hand movement from electrode 2; (c) TI hand movement from electrode 1; (d) TI hand movement from electrode 2; (e) HO hand movement from electrode 1; (f) HO hand movement from electrode 2; (g) PA hand movement from electrode 1; (h) PA hand movement from electrode 2; (i) SP hand movement from electrode 1; (j) SP hand movement from electrode 2; (k) LA hand movement from electrode 1; (l) LA hand movement from electrode 2.

The sEMG signal data set used in this work is obtained from the following publicly available accessible URL: https://archive.ics.uci.edu/ml/datasets/sEMG+for+Basic+Hand+movements [10]. The signals were taken from two differential sEMG sensors, and the signals were transmitted to a 2-channel sEMG system by Delsys Bagnoliâ handheld sEMG systems. There were two forearm sEMG electrodes (flexor capri ulnaris and extensor capri radialis, longus and brevis) held in place by elastic bands and the reference electrode in the middle, in order to gather information about the muscle activation. The sEMG signal data set was collected from 5 healthy subjects (2 males and 3 females; age: 20-22 years). The sampling frequency of the sEMG signal is 500 Hz. The signals were band-pass filtered using a Butterworth Band Pass filter with low and high cutoffs at 15 Hz and 500 Hz, respectively, and a notch filter at 50 Hz to eliminate line interference artifacts. The subjects were asked to perform repeatedly the following six different hand movements: cylindrical (CY), tip (TI), hook (HO), palmar (PA), spherical (SP), and lateral (LA). The force and speed of each hand movement are determined by the subject's willingness, and the recording time of the sEMG signal is 6 seconds. Figure 1 shows the six hand movements used in this work. Each subject repeats these hand movements 30 times. More details of the data set are introduced in the research [53–55].  Figure 2 shows the sEMG signal samples analyzed in this work.

### 3.2. Methods

**Figure 3:**
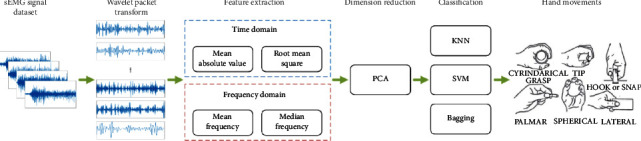
The framework of hand movement classification proposed in this work.

To improve the classification performance of hand movements based on sEMG signals, we propose a novel method for hand movement classification. In this method, the sEMG signal is decomposed into multiple sub-band signals by WPT; the TD and FD features are extracted from the frequency band signals; the PCA is used to eliminate redundant features; and machine learning is used as a predictive model to achieve the purpose of accurately classifying hand movements. The method proposed in this work is of great significance to the development of human-computer interaction, clinical medicine, and prosthetic control. The flow chart of this work is shown in Figure 3.

#### 3.2.1. Wavelet Packet Transform

The WPT is a sophisticated decomposition algorithm that can subdivide the high-frequency and low-frequency components of a signal [39, 56]. The definition of WPT is as follows.

Assuming that the orthogonal scaling function *φ*(*t*) and the wavelet function *ψ*(*t*) have a two-scale relationship,

(1) $$\begin{array}{c} \varphi \left(t\right)=\sqrt{2}\underset{k}{\sum }h\left(k\right)\varphi \left(2t-k\right)\text{,}\\ \psi \left(t\right)=\sqrt{2}\underset{k}{\sum }g\left(k\right)\varphi \left(2t-k\right)\text{,} \end{array}$$

where *h*(*k*) and *g*(*k*) represent the filter coefficient in multiresolution analysis.

Define the recursive function sequence:

(2) $$\begin{array}{c} w_{2n}=\sqrt{2}\underset{k\in Z}{\sum }h\left(k\right)w_{n}\left(2t-k\right)\text{,}\\ w_{2n+1}=\sqrt{2}\underset{k\in Z}{\sum }g\left(k\right)w_{n}\left(2t-k\right)\text{.} \end{array}$$

When *n*=0, *w*_0_(*t*)=*φ*(*t*) and *w*_1_(*t*)=*ψ*(*t*). The wavelet packet {*w*_*n*_(*t*)}_*n*∈*Z*_ is determined by *w*_0_(*t*)=*φ*(*t*).

Each sub-band signal obtained by WPT will be decomposed into two sub-band signals of high and low frequency by two filters of high frequency and low frequency. Therefore, when the number of decomposition layers is the *n*th layer, the number of sub-band signals is 2*n*. Each layer of sub-band signal contains the entire frequency range of the original signal, which also reflects that WPT is a sophisticated signal analysis method.

#### 3.2.2. Feature Extraction

The feature extraction of sEMG signals is a key step in the classification of hand movements based on sEMG signals [40]. In this work, four features are extracted from the sub-band signals obtained by decomposing the sEMG signal by WPT to classify hand movements [41, 42]. These features were selected on the basis of previous studies that showed their usefulness in distinguishing hand movements based on sEMG signals [2, 43]. The details of the four features are as follows.

The MAV feature is the average value of the absolute value of the sEMG signal amplitude of the segment, which represents the energy of the sEMG signal [23]. The expression of the MAV is as follows:

(3) $$\begin{array}{c} \text{MAV}=\frac{1}{N}\overset{N}{\underset{i=1}{\sum }}\left|x_{i}\right|\text{,} \end{array}$$

where *N* is the window length of the sEMG signal and *i* is the *i*th sample point.

The RMS is a measure of the amplitude of the sEMG signal [23]. RMS is defined as

(4) $$\begin{array}{c} \text{RMS}=\sqrt{\frac{1}{N}\overset{N}{\underset{i=1}{\sum }}x_{i}^{2}}\text{.} \end{array}$$

The MNF is the sum of the product of the sEMG power spectrum and the frequency divided by the total sum of the spectrum intensity [23]. The expression of MNF is

(5) $$\begin{array}{c} \text{MNF}=\frac{\sum_{j=1}^{M}f_{j}P_{j}}{\sum_{j=1}^{M}P_{j}}\text{,} \end{array}$$

where *f*_*j*_ is the frequency of the spectrum at frequency bin *j*, *P*_*j*_ is the sEMG power spectrum at frequency bin *j*, and *M* is the length of the frequency bin.

The MDF is a frequency that divides the frequency spectrum into two regions with equal amplitude [23]. The expression of MDF is

(6) $$\begin{array}{c} \overset{MDF}{\underset{j=1}{\sum }}P_{j}=\overset{M}{\underset{j=MDF}{\sum }}P_{j}=\frac{1}{2}\overset{M}{\underset{j=1}{\sum }}P_{j}\text{.} \end{array}$$

#### 3.2.3. Feature Dimension Reduction

The PCA is a multivariate statistical method that can map high-dimensional space data to low-dimensional space and reduce the redundancy of high-dimensional space data [57]. The core idea of PCA is to analyze the input data and project it in the direction with the least information loss and the greatest variance [49]. The process of PCA dimensionality reduction is as follows: calculate the covariance matrix of the decentralized sample data *X*={*x*_1_, *x*_2_,…, *x*_*n*_}, where *n* is the dimension of the sample data:

(7) $$\begin{array}{c} C=\frac{1}{n}X^{T}X\text{.} \end{array}$$

Calculate the eigenvalues of *C* and the corresponding eigenvectors. Arrange the eigenvectors according to the size of the corresponding eigenvalues, and take the eigenvectors corresponding to the first *k* larger eigenvalues to form a matrix *P*.

Reconstruct the reduced dimensionality data space:

(8) $$\begin{array}{c} Y=PX\text{.} \end{array}$$

The reduced dimensionality data space *P* contains most of the information of the original sample data *X*, which effectively simplifies the modal classification problem.

#### 3.2.4. Classification

In this work, three classifiers, namely, K-nearest neighbor (KNN), support vector machine (SVM), and bagging, are used to classify hand movements. The details of these classifiers are as follows.

*(1) KNN*. The principle of the KNN classifier is to find the K data points closest to a specific sample point in the training set based on a certain distance measurement for a given data set, and then, the label with the most categories in the K samples is used as the label of the final prediction sample [58, 59]. The KNN classifier is a supervised learning algorithm and has excellent performance in various biomedical signal processing applications. In this work, the parameters of the KNN classifier include that the distance metric is Euler distance and the method to determine the label of the sample to be tested is the majority voting method.

*(2) SVM*. The SVM is a supervised learning algorithm based on interval maximization and has the advantages of high computational efficiency and strong generalization ability [48, 60, 49]. The purpose of the SVM is to find the optimal hyperplane to maximize the sample interval of different classes of the hyperplane. For linear inseparable data, the kernel function technology is needed to map the linear inseparable feature vector to the high-dimensional linear separable feature space. In this work, the Gaussian kernel function was selected as the SVM kernel function to classify hand movements.

*(3) Bagging*. Bagging is implemented based on the bootstrap sampling method; that is, random sampling with replacement is performed on a given training set and the obtained *m* subsets of the same size are used as the new training set [53, 61]. Train the basic classification algorithm on these *m* training sets to get *m* models. The classification results of the models are voted on, and the category with the most votes is used as the classification result. Bagging can reduce the variance of the basic classifier to obtain a more stable and accurate classification performance. In this work, the decision tree is selected as the basic classifier of bagging to classify hand movements.

#### 3.2.5. Performance Evaluation

In this work, accuracy, recall, precision, and F1-score (F1) are selected to evaluate the classification performance of different classification models [55]. The equations for these four indicators are given as follows:

(9) $$\begin{array}{c} \text{accuracy}=\frac{TP+TN}{TP+TN+FN+FP},\\ \text{recall}=\frac{TP}{TP+FN},\\ \begin{array}{c} \text{precision}=\frac{TP}{TP+FP},\\ F1=\frac{2\times \text{recall}\times \text{precision}}{\text{recall}+\text{precision}}, \end{array} \end{array}$$

where true positive (*TP*) represents that the classification category of the model and the actual category of the sample are both positive, true negative (*TN*) represents that the classification category of the model and the actual category of the sample are both negative; false negative (*FN*) represents that the classification category of the model is negative but the actual category of the sample is positive; and false positive (*FP*) represents that the classification category of the model is positive but the actual category of the sample is negative.

## 4. Results

### 4.1. Decomposition Performance of WPT

**Figure 4:**
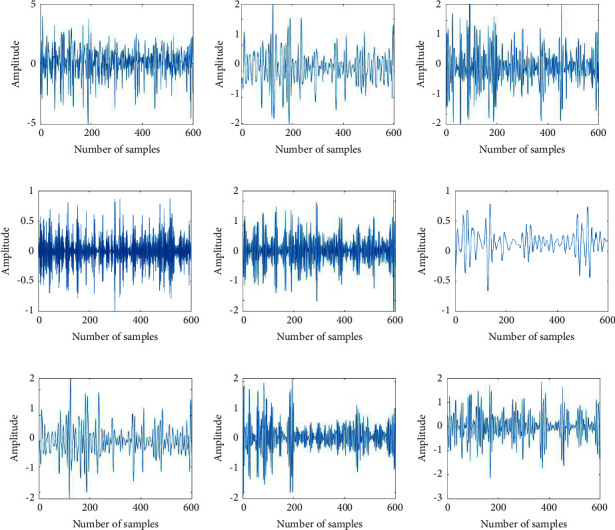
An example of a sEMG signal decomposed by WPT. (a) original sEMG signal; (b∼i) the eight sub-band signals obtained by decomposing the sEMG signal by WPT.

The amplitude of the sEMG signal generated by different hand movements of the same muscle is different. In order to accurately extract the features of the sEMG signal from different hand movements, WPT is used to decompose the sEMG signal. As shown in Figure 4, the sEMG signal is decomposed by the three-layer WPT to obtain the frequency band signal. Figure 4 shows that the waveforms of signals in different frequency bands have unique properties and effectively reflect the intention of hand movements. Therefore, each frequency band signal contains rich features required for hand movement classification.

### 4.2. Wavelet Packet Basis Function Selection

As shown in Table 1, the effect of five different wavelet packet basis functions on the accuracy of hand action classification is evaluated. Combining the classification accuracy of the three classifiers, the classification accuracy of hand movements based on the wavelet packet basis function of dmey is the highest and the classification accuracy of hand movements based on the wavelet packet basis function of sym3 is the lowest. The comprehensive classification accuracy of five different wavelet packet basis functions from high to low is dmey, fk8, coif2, db4, and sym3 (dmey > fk8 > coif2 > db4 > sym3). The combination of dmey wavelet packet basis function and KNN classifier achieves the highest classification accuracy of 97.01%, and the combination of db4 wavelet packet basis function and SVM classifier achieves the lowest classification accuracy of 90.91%. Table 1 shows that the classification accuracy of hand movements based on the dmey wavelet packet basis function is obviously better than other wavelet packet basis functions, and the highest classification accuracy is 97.01%.

### 4.3. Evaluation of sEMG Signal Classification Performance

In order to ensure that PCA reduces the dimensionality of the feature space while ensuring high classification accuracy of hand movements, the classification accuracy of hand movements in five different low-dimensional feature spaces (the extracted feature space is reduced from 64 features to 10, 20, 30, 40, and 50 features) is evaluated.  Table 2 shows the KNN classifier, the SVM classifier, and the bagging classifier achieved 96.03%, 94.50%, and 90.08% classification accuracy in the 30-dimensional feature space, respectively, which is better than the accuracy of the corresponding other-dimensional feature spaces. A comprehensive comparison of the classification accuracy of hand movements in five different low-dimensional feature spaces shows that the 30-dimensional feature space achieves the best classification performance and, combined with the KNN classifier, has the highest classification accuracy of 96.03%.

We also perform statistical analysis of the Kruskal–Wallis test on feature space with dimension 30 (*P* < 0.05 indicates the statistical difference between features) [53]. As shown in Table 3, there is a statistical difference between the 30-dimensional features obtained by PCA dimensionality reduction.

The accuracy, recall, precision, and F1 of each hand movement are calculated to evaluate the classification performance of the proposed method.  Table 4 shows the accuracy, recall, precision, and F1 of each hand movement based on the KNN classifier. The hand movements of TI are classified with the highest accuracy, recall, precision, and F1, the hand movements of CY are classified with the lowest accuracy, recall, and F1, and the hand movements of PA are classified with the lowest precision.  Table 5 presents the classification performance of each hand movement based on the SVM classifier. The hand movements of TI are detected with the highest accuracy, precision, and F1, and the hand movements of HO are detected with the highest recall. On the contrary, the hand movements of HO are detected with the lowest accuracy, precision, and F1, the hand movements of LA are detected with the lowest recall. As shown in Table 6, the hand movements of TI are classified by the bagging classifier with the highest accuracy, recall, precision, and F1, the hand movements of CY are classified by the bagging classifier with the lowest accuracy, precision, and F1, and the hand movements of PA classified by the bagging classifier have the lowest recall. Tables 3–5 show that the classifier has the best classification performance for hand movements of TI and the worst classification performance for hand movements of CY.

## 5. Discussion

In this work, we use WPT to decompose the sEMG signal into multiple sub-band signals to further analyze the intention of hand movements. However, the performance of WPT to decompose the sEMG signal is affected by the wavelet basis function. Therefore, it is necessary to select a suitable wavelet basis function to provide the best classification performance of hand movements. As shown in Table 1, we evaluated the classification accuracy of hand movements of five different wavelet packet basis functions (dmey, fk8, coif2, db4, and sym3). Compared with the wavelet packet basis functions of fk8, coif2, db4, and sym3, the wavelet packet basis functions of dmey have advantages in the classification of hand movement intentions. This can be explained by the research of Shi et al. [56]; that is, the waveform of the dmey wavelet is similar to the sEMG signal, and it has strong compactness and fast attenuation performance. For this reason, the dmey wavelet is capable of analyzing the small change information in the sEMG signal, which is beneficial in improving the classification performance of hand movements.

The MAV, RMS, MNF, and MDF were extracted from sub-band signals obtained from the three-level WPT decomposition sEMG signal. Therefore, the feature space extracted in this work contains 64 features. In order to reduce the dimension of the feature space, the PCA is applied. As shown in Table 2, the accuracy of the feature space of five different dimensions is evaluated. When the feature dimension is 10, the classification accuracy of hand movements is the lowest, which may be caused by the low-dimensional feature space failing to effectively reflect the intention of hand movements [43]. As the dimension of feature space increases, the information of hand movements contained in feature space also increases, which leads to the improvement of the classification accuracy of hand movements [49]. When the dimension of feature space is 30, the classification accuracy of hand movements reaches its highest. However, when the dimension of the feature space exceeds 30, the classification accuracy of hand movements decreases, which can be explained by the reduced classification accuracy caused by the redundancy among features of the feature space with higher dimensions [43]. When the dimension of feature space exceeds 50, the classification accuracy of hand movements may be improved, but the computational complexity also increases. Therefore, a feature space with a dimension of 30 was selected to classify hand movements in this work. In addition, the statistical analysis results in Table 3 also fully prove that the feature space selected with dimension 30 can reduce redundancy among features and retain most information of hand movement intention.

To evaluate the effect of the variability of the sEMG signal on the performance of the proposed model, a robustness analysis is performed on the proposed model. Specifically, different levels of noise are added to the original sEMG signal, and then, the data with noise is processed according to the proposed model in this research. Next, the perturbed data is input into the trained classifier, and the recognition accuracy of hand movements is obtained. Given that the magnitude level of the raw input data is 10^−2^, the corresponding noise level is set to 1 × 10^−2^, 1 × 10^−1^, 3 × 10^−1^, 5 × 10^−1^, and 8 × 10^−1^.  Table 7 shows the robustness results for different noise levels. In Table 7, the proposed method achieves the best hand motion classification performance when the noise level is 1 × 10^−2^ and the hand motion classification performance decreases slightly with the increase of the noise level, which demonstrates that the method proposed in this research has strong robustness. The strong robustness of the proposed method may benefit from the ability of WPT to analyze the intrinsic components of sEMG signals. In addition, Table 7 also shows that the KNN classifier has the best robustness, followed by the SVM classifier and the bagging classifier. The KNN classifier has the strongest robustness, mainly because of the advantage of the KNN algorithm being insensitive to outliers. According to the above robustness analysis, it is concluded that the method proposed in this research has good generalization ability, can accept a wider range of data, and is suitable for real-life applications in the case of unavoidable data disturbances such as electrode displacement and muscle fatigue.

To test the classification accuracy of the proposed method with different window sizes, five tests were performed: 200, 250, 300, 350, and 400 samples. The average classification accuracy of hand movements for different window sizes is shown in Table 8. It can be seen that larger window sizes have better average classification accuracy. This may be attributed to the fact that the larger window size contains more hand motion information. Table 8 shows that the classifier achieves the highest classification accuracy in the case of 400 samples. Although a larger window size may achieve better classification performance, the computational cost of the proposed method is also higher. Considering the classification performance and computational cost, this research analyzes the proposed method based on the window length of 400 samples.

As shown in Table 9, the classification performance of the methods proposed in this work is compared with that of studies performed on the same data set. Akben et al. [14] applied filtering and histogram calculation to the energy values of sEMG signals, and then, the correlation between histogram values was calculated by the consistent correlation method as the features. Their experimental results show that the cascaded-structure classifier achieves the best average classification accuracy of 94.72%. Iqbal et al. [17] proposed a method to classify hand movements from sEMG signals based on singular value decomposition and PCA. They applied singular value decomposition to sEMG signals to extract singular values and the mean and variance of the first five principal components to classify hand movements with an accuracy of 86.71%. Too et al. [32] evaluated the hand movement classification accuracy of sixteen features that were extracted from sEMG signals via discrete wavelet transform. Their results showed that the combination of WL, MAV, enhanced WL, and enhanced MAV achieved an average accuracy of 94.22%. Bergil et al. [57] used the four-level symmetric WT to decompose sEMG signals and calculated the energy, mean value, standard deviation, and entropy of wavelet components as features. The PCA was applied to feature space dimensionality reduction, and the KNN classifier achieved 94.96% average accuracy of hand movement classification. Compared with the existing studies, the proposed method achieves an average classification accuracy of 96.03%, which proves the superiority of the proposed method in the field of hand movement classification.

Inevitably, there are several limitations to this work. First, this work only considers WPT as a sEMG signal decomposition method. In future work, we will apply other popular methods such as empirical mode decomposition and variational mode decomposition to the processing of sEMG signals. Second, this work only classifies six commonly used hand movements, and future work can extend this method to more hand movements. Finally, in the future, we will work on improving the accuracy by improving the proposed method, focusing on tuning and testing for applications in upper limb amputees.

## 6. Conclusions

In this work, we propose a novel method for hand movement classification based on WPT. We also evaluate the effect of WPT based on different wavelet basis functions on hand movement classification, and the experimental results show that the dmey wavelet basis function has the highest classification accuracy. In addition, PCA is used to reduce the dimension of the feature space composed of MAV, RMS, MNF, and MDF to 30 dimensions to achieve high classification accuracy. The KNN classifier was used to classify six kinds of hand movements and achieved an average classification accuracy of 96.03%. Compared with the classification performance of existing research, the proposed method has obvious advantages in classification accuracy. The research results can be applied to exoskeleton robots, rehabilitation training, and intelligent prosthetics [62–65].

## 7. Disclosure

The funders had no role in the design of the study; in the collection, analyses, or interpretation of data; in the writing of the manuscript; and in the decision to publish the results.

## Figures and Tables

**Table 1:** Average classification accuracy of hand movements based on different wavelet basis functions.

Classifier	Wavelet basis function				
	sym3 (%)	fk8 (%)	dmey (%)	db4 (%)	coif2 (%)
KNN	95.91	96.00	97.01	95.94	96.11
SVM	91.08	91.36	92.52	90.91	90.97
Bagging	93.21	94.21	94.89	93.68	93.81

**Table 2:** Average classification accuracy of five different dimensional feature spaces (by using PCA).

Classifier	The dimension of the feature space				
	10 (%)	20 (%)	30 (%)	40 (%)	50 (%)
KNN	90.58	92.72	96.03	94.88	95.95
SVM	92.78	92.01	94.50	88.51	84.38
Bagging	86.66	88.36	90.08	89.34	89.69

**Table 3:** *P* values of 30-dimensional features.

Feature	*P* value
F1	<0.001
F2	<0.001
F3	<0.001
F4	<0.001
F5	<0.001
F6	<0.001
F7	<0.001
F8	<0.001
F9	<0.001
F10	<0.001
F11	<0.001
F12	<0.001
F13	<0.001
F14	<0.001
F15	<0.001
F16	<0.001
F17	<0.001
F18	<0.001
F19	<0.001
F20	<0.001
F21	<0.001
F22	<0.001
F23	<0.001
F24	<0.001
F25	<0.001
F26	<0.001
F27	<0.001
F28	<0.001
F29	<0.001
F30	<0.001

**Table 4:** The classification performance of hand movements obtained by the KNN classifier.

Hand movements	Accuracy (%)	Recall (%)	Precision (%)	F1
CY	98.40	95.23	95.18	0.95
HO	98.73	95.46	96.88	0.96
LA	98.60	95.92	95.70	0.96
PA	98.54	96.46	94.86	0.96
SP	98.695	96.08	96.05	0.96
TI	99.11	97.05	97.58	0.97

**Table 5:** The classification performance of hand movements obtained by the SVM classifier.

Hand movements	Accuracy (%)	Recall (%)	Precision (%)	F1
CY	98.10	93.51	95.02	0.94
HO	96.81	98.87	84.58	0.91
LA	98.27	92.51	96.94	0.95
PA	98.41	92.85	97.50	0.95
SP	98.38	92.82	97.36	0.95
TI	99.03	96.44	97.71	0.97

**Table 6:** The classification performance of hand movements obtained by the bagging classifier.

Hand movements	Accuracy (%)	Recall (%)	Precision (%)	F1
CY	95.17	89.62	82.82	0.86
HO	97.42	92.72	91.89	0.92
LA	96.42	85.67	92.29	0.89
PA	96.68	90.41	89.74	0.90
SP	96.83	88.59	92.08	0.90
TI	97.63	93.46	92.42	0.93

**Table 7:** Robustness results for different noise levels.

Classifier	Noise level				
	1*e* − 2 (%)	1*e* − 1 (%)	3*e* − 1 (%)	5*e* − 1 (%)	8e*e* − 1 (%)
KNN	94.58	94.15	86.61	80.66	76.43
SVM	94.28	93.07	83.65	75.12	69.98
Bagging	89.57	88.41	80.63	73.84	63.92

**Table 8:** Average classification accuracy of hand movements for different window sizes.

Classifier	Window size (samples)				
	200 (%)	250 (%)	300 (%)	350 (%)	400 (%)
KNN	76.58	83.25	88.53	92.14	96.03
SVM	76.34	82.66	87.16	91.28	94.50
Bagging	74.81	79.38	83.60	86.67	90.08

**Table 9:** Comparison of the classification performance of the method proposed in this work with existing studies on the same data set.

Research	Decomposition	Feature	Dimensionality reduction	Classifier	Accuracy (%)
Akben et al. [14]	No	Correlation between histogram values	No	Cascaded-structure classifier	94.72
Iqbal et al. [17]	SVD	Singular values and the mean and VAR	PCA	KNN	86.71
Too et al. [32]	DWT	WL, MAV, EWL, and EMAV	No	NB	94.22
Bergil et al. [57]	Four-level symmetric WT	Energy, mean value, standard deviation, and entropy	PCA	KNN	94.96
Proposed method	WPT	MAV, RMS, MNF, and MDF	PCA	KNN	96.03

## Data Availability

The data used to support the findings of this study is available from the corresponding author upon request.
